# Meek Males and Fighting Females: Sexually-Dimorphic Antipredator Behavior and Locomotor Performance Is Explained by Morphology in Bark Scorpions (*Centruroides vittatus*)

**DOI:** 10.1371/journal.pone.0097648

**Published:** 2014-05-28

**Authors:** Bradley E. Carlson, Shannen McGinley, Matthew P. Rowe

**Affiliations:** 1 Department of Biology, The Pennsylvania State University, University Park, Pennsylvania, United States of America; 2 Intercollege Graduate Degree Program in Ecology, The Pennsylvania State University, University Park, Pennsylvania, United States of America; 3 Department of Biological Sciences, Sam Houston State University, Huntsville, Texas, United States of America; Utrecht University, Netherlands

## Abstract

Sexual dimorphism can result from sexual or ecological selective pressures, but the importance of alternative reproductive roles and trait compensation in generating phenotypic differences between the sexes is poorly understood. We evaluated morphological and behavioral sexual dimorphism in striped bark scorpions (*Centruroides vittatus*). We propose that reproductive roles have driven sexually dimorphic body mass in this species which produces sex differences in locomotor performance. Poor locomotor performance in the females (due to the burden of being gravid) favors compensatory aggression as part of an alternative defensive strategy, while male morphology is coadapted to support a sprinting-based defensive strategy. We tested the effects of sex and morphology on stinging and sprinting performance and characterized overall differences between the sexes in aggressiveness towards simulated threats. Greater body mass was associated with higher sting rates and slower sprinting within sexes, which explained the greater aggression of females (the heavier sex) and, along with longer legs in males, the improved sprint performance in males. These findings suggest females are aggressive to compensate for locomotor costs of reproduction while males possess longer legs to enhance sprinting for predator evasion and mate finding. Sexual dimorphism in the metasoma (“tail”) was unrelated to stinging and sprinting performance and may best be explained by sexual selection.

## Introduction

Sexual dimorphism results from differential selection between the sexes on the same characters, with sex-dependent costs and benefits of traits. The result is sexually divergent phenotypic optima [1]. Biologists have primarily focused on direct sexual selection (via competition for or selection by mates) as the cause of sex differences in body size, ornaments, and weapons, but sexual dimorphism could also result from ecological causes [2]–[4]. In particular, some dimorphisms may serve as adaptations to sex-specific reproductive activities and requirements (the reproductive role hypothesis; [3], [4]). For instance, the additional energy required by females for producing and provisioning embryos (their reproductive role) might explain dimorphisms in trophic structures (mouth or other structures associated with feeding) and other traits associated with energy acquisition and assimilation (e.g. [5]). Under the reproductive role hypothesis, a sexually dimorphic trait that increases fitness in one sex can be expected to have the opposite effect on fitness in the other. For instance, larger heads in female squamates (which permit the consumption of bigger prey) could be expected to have similar growth and maintenance costs for both sexes, but the higher energetic needs of reproduction may outweigh these costs in females [6].

When a sexually-dimorphic trait is costly for its bearers, we expect that secondary characters may develop to compensate for these costs. Trait compensation is well known in nonsexual contexts, and may include both behavioral (e.g. [7]) and morphological compensation (e.g. [8]). Gastropods, for example, exhibit stronger antipredator behavioral responses if they have less effective morphological defenses [7], [9]. Similarly, some sexually dimorphic characters may be the result of compensation for the costs of other traits [10]. Male stalk-eyed flies have larger wings to make up for the flight performance costs of sexually-selected eye stalks [8], and female flying lizards (*Draco melanopogon*) have longer tails and larger ‘wings’ and heads to support gliding performance when encumbered with eggs [11]. In the present study, we explore the potential importance that reproductive roles and compensatory traits may play in morphological and behavioral sexual dimorphism in scorpions.

**Figure 1:**
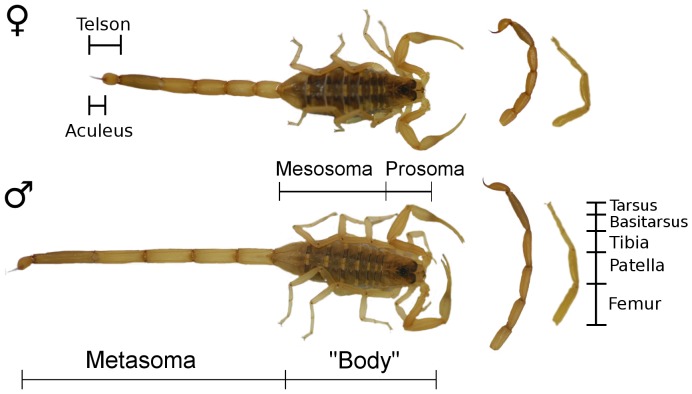
Morphology of representative male and female scorpions (Centruroides vittatus). Whole body, metasoma-alone, and leg-alone (right 4^th^ leg) images are presented on different scales, though relative sex differences are preserved.

Morphological sexual dimorphism is well-documented in many scorpion species (e.g. [12]), but its functional significance and evolution is poorly understood. We focused on overall body size and two poorly understood sexually dimorphic traits that may be related to reproductive roles: metasoma morphology and limb length. Female scorpions are usually larger than the males [13], probably due in part to selection on fecundity and to the direct contribution of developing embryos to body size. In some species (primarily *Centruroides, Isometrus*, *Hadogenes*, and *Urodacus* spp.), males have a slender, elongated metasoma (e.g. [14]–[19]), the so-called ‘tail’ that terminates with the telson (‘sting’; Fig. 1). We know of no studies explaining why male metasomas are sometimes shaped so differently from female metasomas, though it has been suggested that this enables males to identify the sex of conspecifics during courtship [14]. To our knowledge, sexual differences in leg length have not been described in scorpions, but males are typically more mobile while searching for mates [20]–[24] and may have longer limbs to enhance locomotor performance.

Bark scorpions (*Centruroides* spp.) are errant, non-burrowing scorpions [25]–[26], actively moving about on the surface rather than remaining in or near burrows. They therefore rely more heavily on fleeing threats than active defense [27]. We previously reported differences in antipredator performance between male and female striped bark scorpions (*Centruroides vittatus*): males sprint faster than females but tend to deliver stings at a slower rate [15]. The reduced sprinting speed of females is likely caused by the burden of carrying developing embryos while gravid, as female *C. vittatus* sprint faster after the birth and dispersal of their offspring [27] (see also [28] for an example in another taxon). Gravid eight months of the year [29], female *C. vittatus* may be poor at sprinting from predators and might rely more on aggressive defense as a form of behavioral compensation [27] – hence the faster stinging. Male scorpions, on the other hand, are known to be more mobile and range farther than females [20]–[24], exposing them to higher predation risk [23]. Coupled with intrasexual competition to locate receptive females, enhanced locomotor performance should be favored in males [30], and indeed may be easier to achieve without the burden of maternity, and may be the primary antipredator defense.

Sexually dimorphic morphology could also be driven by divergent locomotor capabilities and needs. Bark scorpions exhibit a more slender morphology than more sturdily-built burrowing species, and the males appear to be an exaggerated form of this generalized “errant phenotype” [25]. Long, slender metasomas could therefore be an advantage for sprinting, as they tend to occur in more surface-active species and are usually pronounced in the more active sex, though we know of no comparative studies of sexual dimorphism in scorpions. “Male-like” metasomal morphology might improve locomotor performance in a manner comparable to the positive effects of tails on performance in lizards [31] and crocodilians [32]. Alternatively, shorter, thicker metasomas may be an adaptation to directly support stinging in females, as a comparative study indicated that species with thicker metasomas are more likely to sting defensively [33]. Additionally, longer limbs typically increase stride length and sprinting speed [34], [35] and consequently evasive and mate-finding ability [36], [37]. Females, with little need or capacity for sprinting, may reserve the resources necessary for producing longer limbs for other purposes, resulting in relatively longer limbs in males.

We tested for a relationship between morphology and performance of defensive behavior in both sexes of *C. vittatus*. We characterized sexual differences in the morphology of traits that may contribute to antipredator performance. We also tested whether morphological variation within and between the sexes in dimorphic characters was associated with variation in sprinting and stinging performance. By characterizing the effects of morphology on performance within sexes, this design allowed us to better establish causal relationships of differences between the sexes in both morphology and performance. We predicted that larger, more slender metasomas would support faster sprinting (perhaps at the cost of stinging ability), and that reduced body mass and longer limbs would improve sprinting. We also compared the behavioral responses of males and females to simulated predation threats, with the expectation that females and heavier individuals would respond more aggressively than males and lighter individuals, respectively. Moreover, we anticipated that sex differences in performance and antipredator behavior would be accounted for by morphological differences, in particular the greater mass of females.

## Methods

### Ethics statement

All methods used in this study complied with the requirements of the Institutional Animal Care and Use Committees of Sam Houston State University and The Pennsylvania State University, which regulate animal research at these institutions and do not require protocol reviews or permits for research with invertebrates. This study also complies with relevant state and federal laws. Study subjects were housed with refugia to reduce stress and were provided food and water regularly. All experimental methods caused no apparent injury, and euthanization was performed rapidly by exposure to cold temperatures to minimize any potential pain.

### Study organisms

*Centruroides vittatus* are medium-sized (300v700 mg) lithophilic buthid scorpions, common in the southern United States and northern Mexico [38]. They are predators of smaller invertebrates [24], [26] and are themselves subject to predation by birds, grasshopper mice (*Onychomys* spp.), snakes, lizards, ants, and other scorpions [24], [29], [39], [40]. Scorpions in this study were collected in the Organ Mountains, New Mexico, USA, in July 2011. They were kept at room temperature (25 C) and housed in groups of 100 in plastic sweaterboxes with a gravel substrate, cardboard eggcrates for refugia, and petri dishes for drinking water *ad libitum*. Crickets (*Acheta domesticus*) were provided as food, though scorpions were not fed for several days prior to testing. We did not evaluate the reproductive status of females but note that most appeared to be at some stage of gestation, which is consistent with our previous observations of that >90% of females were gravid in this population at this time of year [15]. However, variation in relative body mass in females is likely substantially affected by the presence and size of the developing brood [27].

### Morphological measurements

We measured the morphology of 30 females and 31 males that had been randomly selected for use in stinging and sprinting trials. Our measurements required scorpions to be euthanized and were thus conducted *after* the trials; however, for clarity we present this information first. We rapidly euthanized the scorpions used in the stinging and sprinting trials by placing them in a freezer for 5–10 minutes. We then separated the mesosoma/prosoma from the metasoma with a razor blade and rapidly weighed each to the nearest milligram. Freezing beforehand prevented body fluids from being lost after cutting, and the sum of the mesosoma and metasoma masses did not differ significantly from the total body mass measured before euthanizing (two-tailed paired t-test: t_59_ = 0.49, p = 0.62).

We photographed each scorpion against a metric ruler, including dorsal photographs of the entire scorpion and lateral photographs of the metasoma and right fourth leg, which was removed by cutting through the trochanter. We used the right fourth leg because one representative leg was desired to simplify analyses and limit multicollinearity. No single leg is consistently used in morphology-performance studies in arachnids [41], [42] and the front legs are often used in scorpion courtship [13] and may thus be dimorphic for non-locomotory reasons. We therefore deemed the fourth leg to be an appropriate choice for this study. Leg photographs were taken after specimens had been stored frozen for approximately six months. While long-term freezing may have distorted the morphology, the initial photographs of the scorpions were unsuitable for proper measurement of the legs, as the legs were not oriented consistently with respect to the camera; we expected that relative differences among individuals would persist. We used ImageJ to take morphological measurements from the photographs, using the ruler in the photographs for scale [43]. We measured the total combined prosoma and mesosoma length (mouth to base of first metasomal segment; hereafter, “body length”), total length of the metasoma (base of first segment to the tip of the aculeus), and total length of right fourth leg (proximal end of femur to distal end of tarsus).

We tested for sexual dimorphism in morphological traits that we anticipated *a priori* may influence the performance of antipredator behavior. We specifically compared “body” (prosoma and mesosoma; Fig. 1) length and mass, metasoma length and mass, relative metasoma thickness, and leg length. We first used a Welch's t-test (correcting for unequal variances) to compare sex differences in body length, which we chose *a priori* as a general body size variable. Morphological characters may simply differ between sexes because one sex is larger. Biologists are therefore interested in whether there are sexual differences in the allometric scaling relationship of traits (i.e the proportional increase in size of one character as another – such as overall size – increases, measured as the slope of one morphological character against another). In particular, comparing the elevation (i.e. intercept) of the scaling relationship between traits tests whether a trait is larger in one sex when holding the other trait (e.g. overall size) constant, provided both sexes have the same slope. We therefore used standardized major axis (SMA) regression to compare the slopes and elevation (i.e. intercepts) of the scaling relationship between body length and other morphological traits to provide a comprehensive description of the nature of sex differences in morphology. SMA regression is preferred for comparing slopes between groups in allometric analyses, as well as testing whether a slope equals a particular value [44]. Diagnostic plots of residuals revealed that the models were properly specified, and all traits (except relative metasoma thickness) were log-transformed prior to analysis [44]. In all cases, there was no significant sex difference in slopes (all p>0.10), so we only considered differences in elevation when describing sexual dimorphism in this species. We present a full description of allometric scaling relationships in Table S1.

### Antipredator behavior performance

We measured stinging performance (rate of stinging and latency to deliver first sting) and sprint speed in a very similar manner to that used in a previous study [15]. We summarize the methods here and present them in full detail as supplementary information in Appendix S1. In stinging trials, scorpions were confined by a transparent cylinder through which they were video-recorded. A probe (stick with a 5 cm^2^ target on the end) was pressed onto each scorpion's back from above, and held in place for 2 s or until the scorpion stung it. This was repeated for three consecutive trials. Scorpions that did not sting in any of the three trials were not used to analyze sting latency or rate, as it was assumed *a priori* that 2 s was a sufficient length of time for a motivated scorpion to sting. We used frame-by-frame analysis of the videos of stinging trials to determine, for each individual scorpion, the maximum sting rate (number of stings delivered in the 2 s following the first contact of the stinger with the target) and minimum sting latency (time from probe contact to stinging the target). We also documented several variables that may influence stinging performance for inclusion as covariates in the analyses: degree of metasomal curling (1–7 scale; [15]), which can increase latency; accidental ‘self-stinging’ rather than hitting the target, which would decrease sting rate and increase latency; and whether the stinger became momentarily stuck in the target, which would decrease sting rate. We tested 30 females and 39 males. Fourteen scorpions (all males) did not sting, resulting in the use of 25 male scorpions for sting rate and latency analyses. As a measure of defensive aggression, we also recorded the number of trials (out of three) in which each scorpion stung the target; for this we included the 14 male scorpions that failed to sting, resulting in a final sample size of 30 females and 39 males for this variable.

Sprint speed was measured by prodding scorpions to run down a sand-covered track until they stopped or had sprinted 50 cm (see Appendix S1 for detailed description of methods). Each scorpion was given two consecutive trials. The time and distance were recorded and the faster speed of the two trials was considered the maximum sprint speed. We used 30 females and 30 males in these tests. In only four cases (all females), the scorpions stopped short of 50 cm.

We used generalized linear models (GLMs) to test for the effects of sex alone, and of all morphological traits together, on stinging and sprinting performance. Multicollinearity was problematic in each analysis involving morphology because metasoma thickness, length, and mass were naturally highly correlated with each other, yielding unacceptably high variance inflation factors (VIF>10; [45]). We thus excluded metasoma length as an independent variable in all analyses because it was strongly correlated with metasoma thickness and mass, whereas these two variables could both be included in all models without multicollinearity issues. Exploratory analyses using metasoma length showed it had similar effects on performance as metasoma mass (without allowing us to independently assess the contribution of metasoma thickness.) We also examined whether sex affected defensive behavior performance when morphological covariates were included. However, metasoma thickness was highly sexually dimorphic and could not be included in the same models as sex. We therefore conducted the analyses using metasoma thickness only, although using sex instead of metasoma thickness yielded qualitatively similar results in all instances.

For sting rate, we used GLMs with a Poisson error distribution and log-link because the response variable was measured as a count (number of stings delivered in 2 s). In the model with morphological variables, we also included as covariates metasoma curling (1–7 scale), whether a ‘self-sting’ occurred (0 or 1), and whether the aculeus ‘stuck’ the target (0 or 1). We excluded leg length from sting speed analysis because we did not hypothesize any effect on stinging ability. We used GLMs with a normal error distribution and identity link (i.e. standard linear regression) to assess the effects of sex and morphology on sting latency and sprint speed. For sting latency, we first tested for overall differences between the sexes with a Welch's t-test, and then used a GLM with body mass, metasoma mass, and metasoma thickness as predictors, along with the covariates of metasoma curling and ‘self-stinging’. Sting latency was log-transformed prior to analyses to meet model assumptions. For sprint speed, we used a Welch's t-test to compare overall performance of males and females, and then used a GLM with body mass, metasoma mass, metasoma thickness, and leg length to test the independent effects of each variable. As before, all morphological variables (except metasoma thickness) were log-transformed prior to analysis.

For each of the above analyses of performance, we also examined a model that included interactions between sex and each morphological variable (omitting metasoma thickness) to determine whether the effects of morphology differed between the sexes. In all cases there were no significant interactions (sting rate [likelihood ratio test, LRT]: χ^2^_2_ = 3.49, p = 0.17; sting latency [ANOVA]: F_2,47_ = 0.96, p = 0.39; sprint speed [ANOVA]: F_3,51_ = 1.33, p = 0.28). We therefore excluded interactions from all final analyses.

### Assays of temperament

Whether a scorpion could be induced to sting during the sting speed trials serves as a plausible measure of aggression, but we devised an additional assay to supplement and support these data. The complementary assay was performed on a separate subset of scorpions. Beginning September 8 2011, this group of scorpions was segregated by sex and housed at similar densities as described earlier in an environmental chamber set at 29 C. Nine male and 12 female scorpions were then transferred to individual containers (measuring 15 cm by 11 cm) a week before behavioral trials (February 13 2012). Each scorpion was then tested once on each of 3 consecutive days. All trials were conducted during the day (between 1000 and 1700 hours) over a one-hour period by a single investigator. Individual scorpions were grasped by the first metasomal segment (base of the ‘tail’) with metal forceps and held approximately 15 cm above the individual housing container for 10 s. The experimenter noted whether the scorpion attempted to sting the forceps during this period and then returned the scorpion to its container.

We used the number of trials in which stings were attempted during the sting speed trials and the temperament assay as measures of aggression. For both tests we categorized the scorpions into two groups: those that never stung or stung in only one trial, and those that stung in either two or all three trials. We tested for associations between sex and the number of trials in which stings occurred with separate Fisher's exact tests for each assay.

All analyses were performed in R version 2.15.1 [46] as two-tailed tests with alpha = 0.05 level of significance. We used the package “smatr” for SMA regression analyses as described in Warton et al. [44]. The dataset can be acquired from The Pennsylvania State University ScholarSphere repository (https://scholarsphere.psu.edu/files/6395w957h) or by contacting the corresponding author directly.

## Results

**Figure 2:**
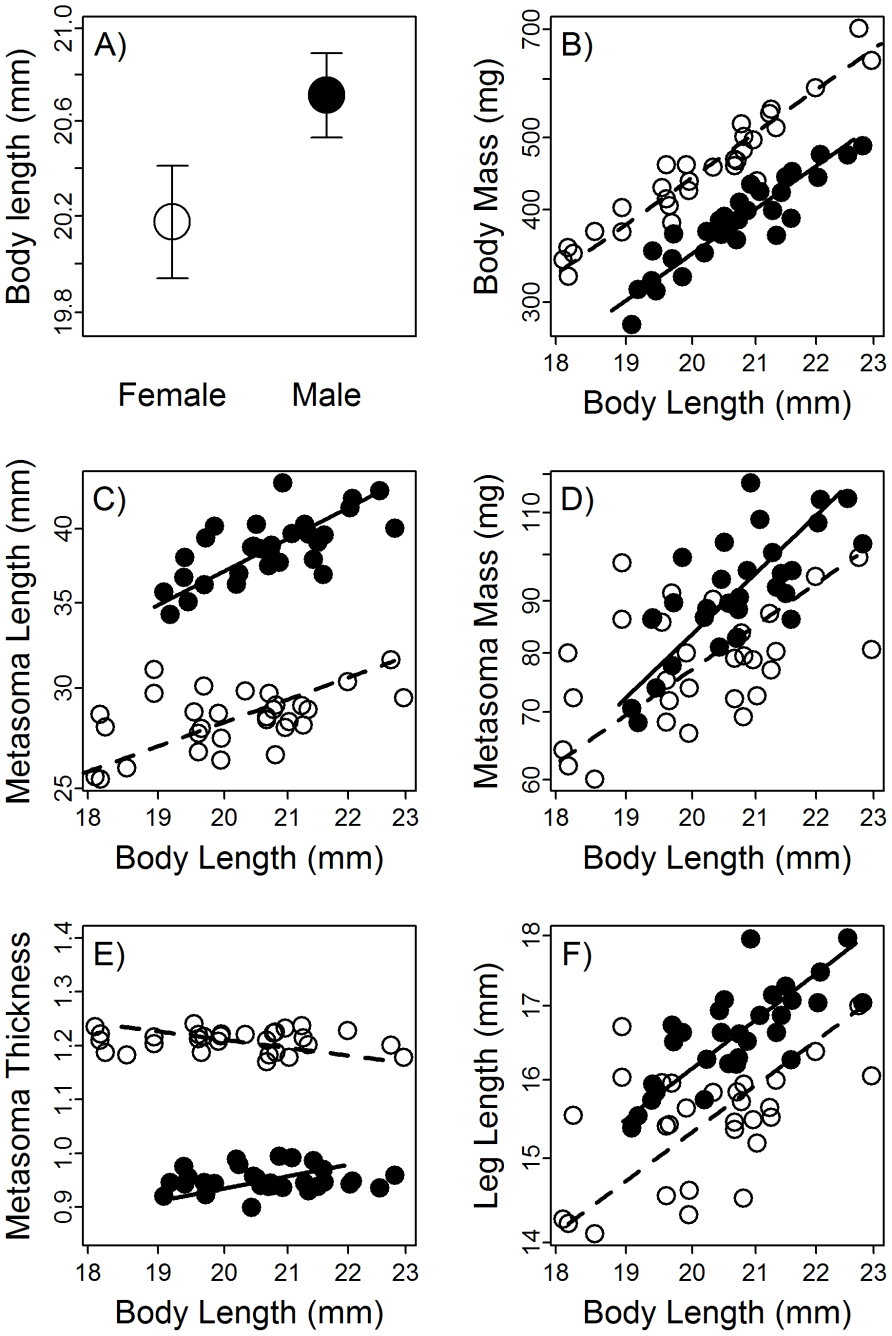
Morphological differences between female (open circles) and male (filled circles) *C. vittatus* scorpions. B–F depict allometric scaling relationships of traits with body length, and lines represent the fitted relationship within each sex. Metasoma thickness (E) was calculated as metasoma mass (transformed by the inverse-power of its scaling relationship with length) divided by metasoma length. Scales are all logarithmic, except for relative metasoma thickness.

Male scorpions tended to have longer bodies than females, but the difference was marginally non-significant (t_53.80_ = 1.86, p = 0.07; Fig. 2A). For a given body length, body mass was generally higher in females (χ^2^_1_ = 252.3, p<0.0001; Fig. 2B) and metasoma length was higher in males (χ^2^_1_ = 387.6, p<0.0001; Fig. 2C). Males also had heavier metasomas (χ^2^_1_ = 7.5, p = 0.006; Fig. 2D) that were relatively thinner than the females’ (χ^2^_1_ = 1009.0, p<0.0001; Fig. 2E). Limb length was also longer in males (χ^2^_1_ = 24.18, p<0.0001; Fig. 2F).

Female scorpions stung at a significantly faster rate overall (8.0±0.97 stings [per 2 s], mean ± one standard error) than males (6.5±0.62 stings; p = 0.034; Table 1). Body mass was positively associated with sting rate (p = 0.003), but no other morphological variables (or sex itself) had significant independent effects on sting rate (all p>0.33; Table 1). No morphological variables affected sting latency independently (all p>0.39), but males tended to have longer sting latencies (0.54±0.14 s) than females (0.31±0.06 s), though the difference was not significant (p = 0.14; Table 1). Overall, males sprinted faster (22.4±0.67 cm/s) than females (17.6±0.98 cm/s; p = 0.0002; Table 1). This was explained by males having lighter bodies and longer legs, both of which were associated with faster sprinting (p<0.05), without any significant effects of metasoma mass or metasoma thickness (the latter is again qualitatively identical to the effect of sex; p>0.10; Table 1).

**Table 1:** Effects of sex and morphology on stinging and sprinting performance. For each effect, the table depicts p-values, test statistics, and (for all p≤0.10) direction of effect.

Performance measure	Sex (overall)^a^	Body mass	Metasoma mass	Relative metasoma thickness ( = Sex)^b^	Leg length	Overall model^c^
Sting rate	**p = 0.034**	**p = 0.003**	p = 0.44	p = 0.57	n/a	**p = 0.006**
	**χ^2^_1_ = 4.47**	**χ^2^_1_ = 8.69**	?^2^_1_ = 0.59	?^2^_1_ = 0.32	n/a	**χ^2^_6_ = 18.1**
Sting latency	p = 0.27	p = 0.39	p = 0.51	p = 0.94	n/a	p = 0.095
	T_43.0_ = −1.12	t_49_ = −0.88	t_49_ = 0.66	t_49_ = 0.08	n/a	F_5,49_ = 2.0
Sprint speed	**p = 0.0002**	**p<0.0001**	p = 0.34	p = 0.10	**p = 0.046**	**p<0.0001**
	**t_51.18_ = −4.04**	**t_54_ = −4.38**	t_54_ = 0.96	t_54_ = 1.67	**t_54_ = 2.04**	**F_3,54_ = 12.5**

a Not controlling for effects of covariates.

b The effects of relative metasoma thickness are qualitatively the same as sex.

c Overall model includes additional control covariates for sting rate and latency (see *Methods*).

Females were more likely to attack simulated threats during both the sting speed trials and the temperament assays (Table 2). In fact, every female stung at least once during the sting speed trials, while only 64% of males did (Table 2a). Similarly, no males attempted to sting at all during the temperament assays, while 75% of females stung in at least one trial (Table 2b).

**Table 2:** Numbers of males and females which stung no more than once or at least twice during a) sting speed trials, and b) temperament assays.

a) Number of trials scorpion stung	Males	Females	Fisher's exact test
≤1	20	3	p = 0.0003
≥2	19	27	

b) Number of trials scorpion stung	Males	Females	Fisher's exact test
≤1	9	5	p = 0.007
≥2	0	7	

## Discussion

Striped bark scorpions (*Centruroides vittatus*) are morphologically and behaviorally sexually dimorphic, and variation in some traits was related to stinging and sprinting performance. Female scorpions have heavier bodies, shorter legs, and metasomas that are lighter, shorter, and thicker. Female scorpions also stung at faster rates, tended to have shorter sting latencies (though this difference was not significant), sprinted slower, and were more likely to attack a simulated threat. The sex difference in sprinting performance was accounted for by greater body mass in females, as expected based on previous work in this [27] and other species (e.g. [47]). Interestingly, heavier bodies were also associated with increased sting rates, again both across and within sexes. No aspect of metasoma morphology was related to either stinging or sprinting, contrasting with interspecific patterns in scorpions in which thicker metasomas are associated with defensive stinging [33]. However, longer limbs increased sprint speed, and the longer legs of males, in addition to their lighter bodies, appear to be the key to their nearly 30% increase in sprint speed over females. Sex (which was qualitatively equivalent to relative metasoma thickness in the analyses) was not a significant predictor of sting rate or sprint speed when morphological traits were included in the analysis, and the effects of morphology were consistent between the sexes (that is, there were no significant interactions between sex and morphology on any of the performance variables). This indicates that the sex differences in these traits were explained by morphology, suggesting that sexually dimorphic antipredator behavior may be mediated by morphological variation.

While shorter legs and the additional burden of a heavy body easily explain the reduced locomotor performance of females, the relationship between body mass and sting rate is less intuitive. It may be the result of improved mechanical or physiological support for stinging, such as enhanced energy reserves, greater musculature in the posterior mesosoma, or increased capacity to generate internal hydrostatic pressure. Alternatively, the relationship could be caused by behavioral compensation for reduced sprinting ability. The latter seems more likely, as we also demonstrated a greater propensity for females to attack perceived threats in two different contexts. Greater aggression when the sprinting ability of reproductive female bark scorpions is most diminished has been previously reported [27], and our work thus extends this negative relationship between sprinting and stinging to behavioral differences between the sexes. Behavioral compensation for higher vulnerability appears to be a common phenomenon in both reproductive (e.g. [48], [49]) and non-reproductive contexts (e.g. [7], [50], [51]) in other species. The males were surprisingly reticent to sting, despite what we interpreted as clear indications of mortal danger (suddenly being pressed upon from above, and being lifted). The behavioral assays we used may be sufficiently different from natural predation events that they failed to elicit a response. However, we observed a bark scorpion that remained immobile and did not sting a predatory alligator lizard (*Elgaria kingii*) as it walked over the scorpion in a staged event (M.P. Rowe, pers. obs.), indicating that scorpions may strategically refrain from stinging. In addition, other scorpion species have been documented exhibiting minimal responses to simulated threats [33]. Given the significant metabolic expense of venom production and the time needed for venom to return to peak levels of potency [52], [53], it is perhaps unsurprising that scorpions would sometimes avoid stinging if other options (e.g. fleeing) are available. Aggression may also have been reduced due to acclimation to (or the stressful effects of) captive conditions. However, males and females were treated identically suggesting that there is indeed a sexual difference in aggression, though the average level of aggression may have been altered due to an experimental artifact.

We did not detect any effects of metasoma mass or relative thickness on any measure of performance. Though a heavier metasoma should decrease sprint speed due to the increased mass and friction [54], we hypothesized that its presence in males may indicate that it increases performance. Metasoma mass did not impact sprinting performance, which suggests that it may in fact have some positive effects on sprinting that balance the cost of the additional mass. Males are therefore capable of producing larger metasomas without an associated decrease in sprinting performance. But why do males possess larger metasomas if they do not enhance locomotion? Or, why do females possess smaller metasomas if they don't improve stinging ability?

Metasomas may assist in other aspects of locomotion beyond straight-line sprinting. Though maneuverability can be negatively impacted by the increased rotational inertia produced by tails [55], lateral movements of a tail can potentiate rapid turns in insect-like robots [56]. In this case, we might hypothesize that females do not invest in a longer metasoma because their minimal sprinting capabilities favor diverting resources to other structures or to offspring. Alternatively, this dimorphism may be the result of sexual selection. Male metasomas were not as dramatically heavier than females' as they were longer and thinner (Fig. 2C–E), suggesting that selection is targeting longer metasomas while minimizing the increase in mass. The thinness of the male metasoma may reduce the weight and hence the locomotor performance costs of this putatively sexually-selected trait [57], permitting males with long metasomas to sprint with only limited impairment. Longer metasomas may increase the “reach” of males, better enabling them to deflect sting attempts by resistant females during courtship, deliver sexual stings [58], and/or combat other males. Little is known about sexual selection in scorpions in general as well as in this species, so these hypotheses are presently difficult to assess. Comparative studies of the use of the metasoma in courtship by sexually dimorphic and monomorphic species could provide valuable insight. It is worth noting that there was a nonsignificant tendency (p = 0.11; Table S1) for male scorpions to have steeper allometric slopes for metasoma length (Fig. 2C; see also [18] for similar findings in a closely related species). It has been frequently argued that it is a hallmark of sexual selection when one sex exhibits hyperallometry (disproportionately high increases in size of a trait as body size increases; [59]) but the reliability of this relationship has been seriously questioned [60]. If sexual selection has favored greater metasoma length in males in some scorpions (such as *Centruroides* spp.), why not in others? One intriguing, albeit speculative, possibility is that metasoma morphology is less evolutionarily labile in burrowing species which may require stout metasomas for digging activity. Some scorpion species have been described using the metasoma as a brace while digging [61] and to push loosened soil out of the burrow [62].

Though the evolutionary origins of metasoma dimorphism in scorpions remains enigmatic, we have shown that female *C. vittatus* exhibit poor sprinting ability (due to higher body mass) and that they appear to compensate by stinging more rapidly and readily. This is consistent with the overall image of female scorpions being less mobile and active while males, unburdened by parenthood, are effective sprinters that have evolved longer legs to support predator evasion and mate-seeking [21]–[24], [36]. Further research, especially in non-traditional study organisms such as scorpions, will further illuminate the complex ecological causes and consequences of the evolution of sexually dimorphic characters.

## Supporting Information

Appendix S1 **Expanded description of methods for measuring scorpion stinging and sprinting performance.**
(DOC)

Table S1 **Summary of tests on allometric scaling relationships of morphological traits with body length and sex differences in allometric elevation.** Estimated allometric slopes for each sex are presented with 95% confidence intervals below. Test statistics and p-values are derived from likelihood ratio tests (LRT) performed in standardized major axis regression as described in [44], and all traits except relative metasoma thickness were log-transformed for analysis. Mass is expected to increase as a function of the third power of linear measurements, so isometry is indicated by a slope of three on the (log-transformed) mass variables. Relative metasoma thickness was calculated as metasoma mass divided by metasoma length, with mass transformed according to its empirical scaling relationship with length (2.38th -root transformed; see below). Because metasoma thickness is already relative to length, isometry here is indicated by a slope of 0.
(DOC)
