# Supplementary material for: Meek Males and Fighting Females: Sexually-Dimorphic Antipredator Behavior and Locomotor Performance Is Explained by Morphology in Bark Scorpions (Centruroides vittatus)
Source: PLoS One. 2014 May 28;9(5):e97648. doi: 10.1371/journal.pone.0097648 (PMC4037197; doi:10.1371/journal.pone.0097648)
Supplement: Appendix S1 — Expanded description of methods for measuring scorpion stinging and sprinting performance. (DOC) [file pone.0097648.s001.doc]

Appendix S1. Expanded description of methods for measuring scorpion stinging and sprinting performance.

*Stinging performance.* Sting rate and latency were measured using methods similar to those previously described in [15]. Sting trials were conducted on a copper plate partially covered with a 0.5 cm deep layer of play sand. A laterally-compressed cylinder of transparent acetate sheet, measuring approximately 7 cm long x 3 cm wide x 8 cm tall, rested in the sand and served to contain the scorpions and restrict their movements. A Canon XL2 digital video camera was set up facing the long side of the acetate loop. Scorpions were placed in the acetate loop at the beginning of each trial. The position of the loop and angle of the copper base plate were adjusted as needed to ensure the scorpion was perpendicular to the camera view. A probe constructed from a 15 cm stick with a 2 x 2.5 cm cardstock target on the end was then pressed onto the anterior mesosoma (“abdomen”; Fig. 1), eliciting defensive stings directed at the target. A single experimenter performed each trial to maintain consistency in the speed and pressure with which the probe was used. The trial was terminated 2 seconds (s) after the first sting or, if the scorpion failed to respond, after 2 s of pressing with the probe. Consecutive second and third trials were performed with several seconds in between each trial. We determined *a priori* that a failure to sting within 2 s likely represents differences in motivation rather than capacity to sting, as all scorpions were healthy and active enough that it was reasonable to expect they should be able to sting within this time if so desired. Scorpions that did not sting within 2 s in any trials were typically replaced with new scorpions of the same sex; however, five male scorpions were discovered to have not stung successfully only after review of the video recordings and hence were not replaced at the time of the trial, resulting in a total of 25 stinging males. The room temperature remained 25 C for the duration of these trials. We alternated male and female trials to minimize potential bias associated with subtle changes in conditions over the course of the experiment.

Trials were video-recorded at 30 frames s^-1^ and subsequently analysed with Media Player Classic version 1.3.2121.0 (available from http://sourceforge.net/projects/mpc-hc/, accessed 26 July 2010). We characterized three response variables: sting rate, sting latency, and number of attacks. Sting rate was calculated as the number of stings (tip of aculeus touching the target) delivered in the first 2 s (60 frames) following contact between the probe and the scorpion. We used the highest sting count for each individual across the three trials as the scorpion’s sting rate. We measured sting latency as the time elapsed from the probe contacting the scorpion to the delivery of the first sting to the target. We used the shortest latency among the three trials for each scorpion in the analysis, excluding scorpions that did not sting within 2 s in any of their trials. Finally, the number of attacks was the total number of trials (0-3) in which the scorpion stung the target within 2 s of contact by the probe. For this metric, we included scorpions that did not sting in any trials and were replaced, resulting in a final sample size of 30 females and 39 males. During each trial, we also documented several covariates that may influence sting rate and/or latency. Because it may influence stinging performance, the degree of curling of the metasoma immediately prior to probe contact was scored on a 1-7 scale (see Figure 2A in [15]). Additionally, the aculeus sometimes inadvertently first made contact with the dorsal surface of the scorpion’s mesosoma, sometimes causing the scorpion to pause briefly while presumably assessing the efficacy of its strike. We therefore noted whether or not a ‘self-strike’ occurred as we expected these misdirected stings to increase latency. Finally, we also noted whether the aculeus stuck into the cardstock target during any of the stings; this was observed as the aculeus striking the target and the tip remaining stationary as the metasoma continued to move as though trying to dislodge the sting. We anticipated scorpions who ‘stuck’ the target would have a lower sting rate.

*Sprinting performance.* Sprinting trials were conducted in a 122 cm x 35 cm track constructed of a copper baseplate and aluminium sides, divided lengthwise into smaller interior tracks measuring 10.5 cm in width [15]. A metre stick was placed in the track to enable sprint distances to be measured. The substrate was an approximately 1 cm deep layer of play sand that was smoothed between trials to maintain an even and consistent running surface. One end of the track was brightly lit from above with a fluorescent lamp while the other end was shaded to encourage the scorpion to run in the proper direction, as they exhibit strong negative phototaxis. The trials were all conducted during the day (between 1000 and 1800 hours) at a constant controlled room temperature of 25 C, and again males and females were alternately tested.

At the beginning of each trial, the scorpion was placed in a small transparent acetate cylinder (approximately 6 cm diameter) sunk into the sand on the brightly lit end of the track and positioned at the zero mark of the metre stick. If necessary, the scorpion was repositioned within the cylinder so that it faced the dark end of the track. After a brief period during which the trial was prepared (< 1 minute), the cylinder was removed and the scorpion was tapped on the metasoma to initiate running. A digital stopwatch was used to measure the elapsed time from the moment the scorpion began running to either 1) the scorpion's head crossed the 50 cm mark, or 2) the scorpion stopped sprinting. If the scorpion stopped short of 50 cm, the distance actually run was recorded. Scorpions sprinted unidirectionally in all cases, with no individuals walking or otherwise moving distinctly slower over one portion of the track. Individuals that stopped short typically halted suddenly. After the trial ended, the scorpion was returned to the cylinder at the start of the track, the sand was smoothed, and a second trial was conducted in the same manner as the first. In the analyses, sprint speed was calculated as cm/s and the faster of the two trials was used as the maximum sprint speed. In four cases (all females), the maximum sprint speed was measured over less than 50 cm (31, 37, 40, and 48 cm). The use of two trials is supported by previous work in which we found that 96 % of bark scorpions sprinted fastest in their first or second trial of three (B.E. Carlson & M.P. Rowe, unpublished data).
