# Supplementary material for: Meek Males and Fighting Females: Sexually-Dimorphic Antipredator Behavior and Locomotor Performance Is Explained by Morphology in Bark Scorpions (Centruroides vittatus)
Source: PLoS One. 2014 May 28;9(5):e97648. doi: 10.1371/journal.pone.0097648 (PMC4037197; doi:10.1371/journal.pone.0097648)
Supplement: Table S1 — Summary of tests on allometric scaling relationships of morphological traits with body length and sex differences in allometric elevation. Estimated allometric slopes for each sex are presented with 95% confidence intervals below. Test statistics and p-values are derived from likelihood ratio tests (LRT) performed in standardized major axis regression as described in [44], and all traits except relative metasoma thickness were log-transformed for analysis. Mass is expected to increase as a function of the third power of linear measurements, so isometry is indicated by a slope of three on the (log-transformed) mass variables. Relative metasoma thickness was calculated as metasoma mass divided by metasoma length, with mass transformed according to its empirical scaling relationship with length (2.38th -root transformed; see below). Because metasoma thickness is already relative to length, isometry here is indicated by a slope of 0. (DOC) [file pone.0097648.s002.doc]

Table S1. Summary of tests on allometric scaling relationships of morphological traits with body length and sex differences in allometric elevation. Estimated allometric slopes for each sex are presented with 95% confidence intervals below. Test statistics and p-values are derived from likelihood ratio tests (LRT) performed in standardized major axis regression as described in [44], and all traits except relative metasoma thickness were log-transformed for analysis. Mass is expected to increase as a function of the third power of linear measurements, so isometry is indicated by a slope of three on the (log-transformed) mass variables. Relative metasoma thickness was calculated as metasoma mass divided by metasoma length, with mass transformed according to its empirical scaling relationship with length (2.38^th^ -root transformed; see below). Because metasoma thickness is already relative to length, isometry here is indicated by a slope of 0.

| Trait | Female allometric slope | Male allometric slope | Sexual difference in allometric slope | Deviance from isometric slope | Sexual difference in allometric elevation |
| --- | --- | --- | --- | --- | --- |
| Body mass | 2.86  (2.55 – 3.21) | 2.85  (2.44 – 3.33) | p = 0.98  χ^2^_1_ = 0.0007 | p = 0.57  χ^2^_2_ = 1.12 | **p < 0.0001**  χ^2^_1_ = 252.3  (**♀ > ♂**) |
| Metasoma length | 0.85  (0.61 – 1.19) | 1.20  (0.91 – 1.59) | p = 0.11  χ^2^_1_ = 2.54 | p = 0.26  χ^2^_2_ = 2.66 | **p < 0.0001**  χ^2^_1_ = 387.6  (**♂ > ♀**) |
| Metasoma mass | 2.03  (1.45 – 2.86) | 2.82  (2.17 – 3.67) | p = 0.13  χ^2^_1_ = 2.29 | **p < 0.0001**  χ^2^_2_ = 55.06  (Hypoallometric) | **p = 0.006**  χ^2^_1_ = 7.51  (**♂ > ♀**) |
| Relative metasoma thickness | -0.007  (-0.01 –  -0.005) | 0.011  (0.007 – 0.016) | p = 0.12  χ^2^_1_ = 2.39 | **p < 0.0001**  χ^2^_2_ = 922.3  (Hypoallometric) | **p < 0.0001**  χ^2^_2_ = 1009  (**♀ > ♂**) |
| Limb length (4^th^) | 0.78  (0.56 – 1.10) | 0.81  (0.63 – 1.05) | p = 0.87  χ^2^_1_ = 0.03 | p = 0.10  χ^2^_2_ = 4.60 | p **< 0.0001**  χ^2^_1_ = 24.18  (**♂ > ♀**) |
